# The Potential of Double-Faced Polyester-Viscose Woven Fabric as a Porous Substrate for Direct-Coating and Multilayer Concept

**DOI:** 10.3390/polym15234579

**Published:** 2023-11-30

**Authors:** Asril Senoaji Soekoco, Dody Mustafa, Dinan Oktavian, Fahruk Bahtiar, Tina Martina, Nugraha, Brian Yuliarto

**Affiliations:** 1 Department of Engineering Physics, Faculty of Industrial Technology, Institut Teknologi Bandung, Kota Bandung 40132, Indonesia; 2 Department of Textile Engineering, Politeknik STTT Bandung, Kota Bandung 40272, Indonesia; dody-mustafa@kemenperin.go.id (D.M.); dinan@kemenperin.go.id (D.O.); bahtiarfm2505@gmail.com (F.B.); tina-martina@kemenperin.go.id (T.M.)

**Keywords:** biosensor, textile-based sensor, porous substrate

## Abstract

Textile-based sensors fabricated using the direct-coating method are the appropriate choice to meet the aspects of flexibility, non-invasiveness, and lightness for continuous monitoring of the human body. The characteristics of the sensor substrate are directly influenced by factors such as the type of weave, thread fineness, fabric density, and the type of polymeric constituent fibers. The fabric used as the sensor substrate, fabricated using the direct-coating method, must be capable of retaining the electrode paste solution, which has higher viscosity, on one surface of the fabric to avoid short circuits during the fabrication process. However, during its application, this fabric should allow the easy passage of analyte solutions with low viscosity as much as possible. Hence, an appropriate fabric construction is required to serve as the substrate for textile-based sensors to ensure the success of the fabrication process and the effectiveness of the resulting sensor’s performance. The development of the structural design of the fabric to be used as a substrate for non-invasive biosensors with a multilayer concept is carried out by weaving and sewing processes utilizing polyester-viscose fibers. During the production process, variations are applied, such as weft yarn density, the characterization of wetting time, absorption rate, maximum wetted radius, spreading speed, and accumulative one-way transport index. The most suitable fabric for use as a substrate for non-invasive biosensors with a multilayer concept, such as in this research, is a fabric with a weft thread density of 70 strands per inch, along with the addition of an analyte transfer thread configuration.

## 1. Introduction

**Figure 1:**
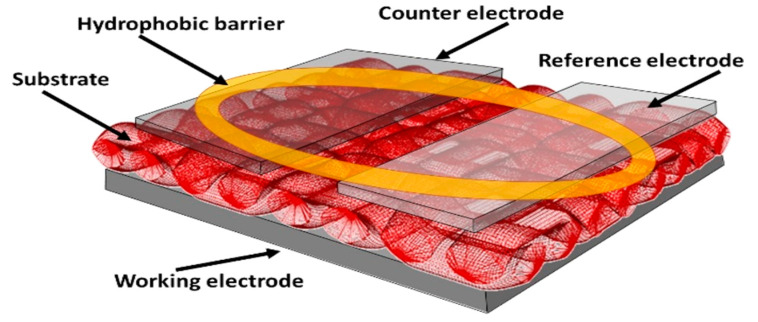
Schematic diagram of multilayer concept.

Health measurement devices, including those for glucose, lactic acid, and cholesterol monitoring, are anticipated to exhibit excellent performance throughout their usage [1]. These devices typically incorporate electrodes that exert a direct influence on sensor performance. Among the critical factors, the surface area of the working electrode holds paramount importance. This surface is coated with an enzyme serving as a catalyst in the oxidation process, facilitating electron transfers and the generation of electrical current [2]. Expanding the surface area of the working electrode results in a larger region for the oxidation process, thereby accelerating the reaction rate and amplifying the electrical signal. A larger electrical signal can enhance measurement sensitivity and accuracy, ultimately leading to improved sensor performance [3]. However, there are constraints on the dimensions of the sensor because it is desirable to keep the sensor as compact as possible. One innovative solution to address this challenge is the utilization of a textile-based sensor as the substrate material. Textile-based sensors possess a porous structure that enables fluid transfer from one side to the other. This feature represents a notable advantage of textile-based sensors as it allows the analyte to be applied to both surfaces of the fabric. This capability facilitates the placement of electrodes across multiple substrate layers, yielding a sensor configuration consisting of a minimum of three layers (upper electrode, substrate, and lower electrode). The depiction of this multilayer concept is presented in Figure 1.

Textile-based sensors created through the direct-coating method are a suitable choice for fulfilling the criteria of flexibility, ease of transportation, non-invasiveness, and lightness. These sensors heavily rely on the design and structure of their substrate, which includes the arrangement of layers and electrode positions. The positioning of the electrodes must be carefully arranged to prevent any contact between them prior to the introduction of the analyte. This precaution is essential to prevent short circuits during the fabrication process. Simultaneously, the sensor should facilitate the easy penetration of low viscosity analyte solutions. This enables the analyte to access all surfaces of the working electrode, counter electrode, and reference electrode. This, in turn, has a direct impact on the minimum amount of analyte required to initiate reduction and oxidation reactions on the sensor, as well as the initial detection time during measurements. Therefore, a well-designed sensor structure is imperative to achieve optimal performance in textile-based sensors employing the multilayer concept. Double-faced woven fabric offers the advantage of a thicker structural design, which serves as a barrier against short circuits during the electrode paste coating process. This fabric has a layered structure composed of two groups of warp threads, causing the upper and lower surfaces of the fabric to be more separated compared to typical fabrics. The configuration of this layered structure on the substrate establishes a moisture barrier that significantly influences the movement of both liquid and vapor between the two fabric surfaces [4,5]. This is expected to effectively retain the electrode paste on one surface of the fabric during the fabrication process [6]. The structural design of a fabric is directly influenced by several factors, and one of them is fabric density, which is determined by the density of the weft threads [7,8,9,10]. In this study, the permeability of a fabric specifically designed as a substrate for a biosensor was controlled by varying the number of weft yarn, with densities of 90 strands per inch, 80 strands per inch, 70 strands per inch, and 70 strands per inch with the addition of analyte transfer thread (ATT). The analyte transfer thread is a rayon viscose thread positioned along the z axis within the fabric, with the objective of expediting the flow of fluid from one side of the fabric to the other [11,12]. All these parameters directly influence the fabric’s porosity and permeability, which determine the sensor performance [13,14]. The determination of fabric permeability utilized as a substrate can be quantified through the fabric’s ability to facilitate fluid flow from one surface to another. This method yields quantitative data, subsequently utilized as a reference for classifying the suitability of fabric types utilized as substrates for biosensor applications. The most suitable fabric for deployment as a biosensor substrate is one characterized by a classification of Moisture Management Fabric and a high Accumulative One Way Transport Index.

## 2. Materials and Methods

**Figure 2:**
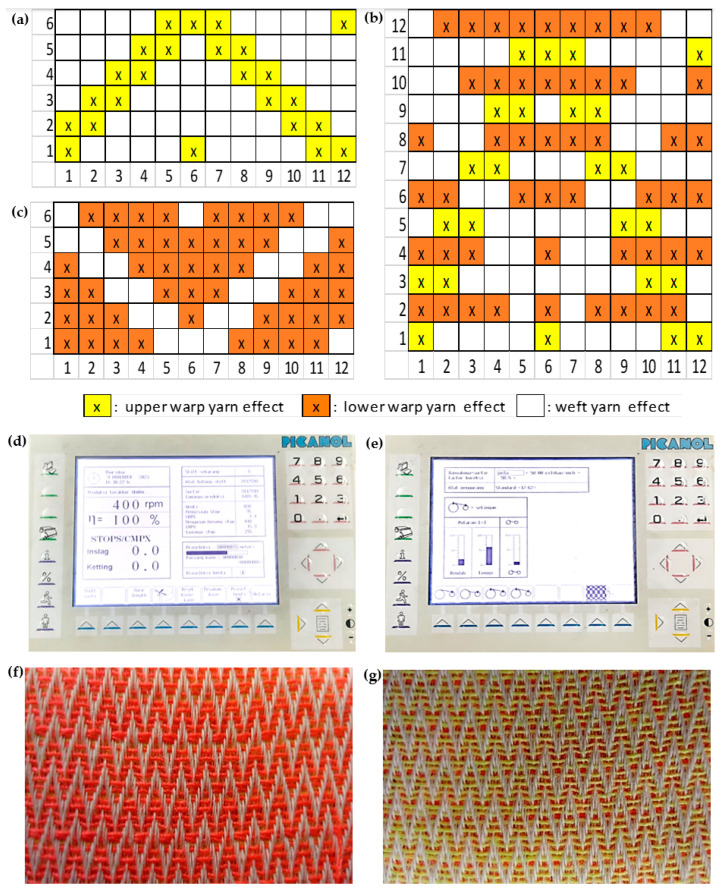
(**a**) Upper face fabric pattern. (**b**) Double-faced fabric pattern. (**c**) Lower face fabric pattern. (**d**) Weaving speed setting. (**e**) Pick density setting. (**f**) Upper face fabric surface. (**g**) Lower face fabric surface.

Polyester–viscose (65: 35) was used as the warp yarn, which was procured from Dhanar Mas Concern in Bandung, Indonesia, while 100% polyester was employed as the weft yarn, sourced from Elephant Star, also in Bandung, Indonesia. The substrate material in this context comprised a blend of polyester and rayon viscose fibers, strategically chosen to attain the utmost spreading radius and wetting speed [15,16]. The incorporation of polyester fibers serves to enhance the fabric’s permeability, owing to the rounded shape and smooth surface of these fibers, which subsequently leads to a reduction in drag resistance [13]. The utilization of hydrophilic rayon viscose low twist threads, characterized by a high moisture regain of 11.5%, proves advantageous in enhancing the absorption capacity. This attribute facilitates the transportation of analytes, allowing them to migrate from one surface of the fabric to another. The design and production of the double-faced fabric in this study were carried out with the assistance of software tools, namely WiseTex version 3.2 developed by Katholieke Universiteit Leuven and TexGen version 3.12.2 developed by University of Nottingham. The double-faced woven pattern design can be seen in Figure 2a–c. The characterization of fabric permeability to liquid fluids was carried out using the Moisture Management Tester instrument (SDL Atlas, Rock Hill, SC, USA), following the AATCC TM 195-2011 standard for assessing the Liquid Moisture Management Properties of Textile Fabrics [17].

### Fabrication of the Double-Faced Woven Fabric

Double-faced woven fabric has a thick structural formation, which can prevent short circuits during the coating process in the sensor fabrication. The substrate employed in this sensor exhibits a layered design structure meticulously arranged to provide optimal permeability. The arrangement of this stratified structure on the substrate creates a moisture barrier that has a substantial impact on the transfer of both liquids and vapors between the two fabric layers [4]. The three-dimensional simulation of a single-layer fabric was conducted with the assistance of the Wisetex application, employing 8 sets of warp and weft yarns. Meanwhile, a three-dimensional simulation of a multilayer fabric was generated using the TexGen application, incorporating 40 strands of warp yarn and 32 strands of weft yarn.

One method employed to regulate permeability involves the incorporation of additional threads oriented perpendicularly to both the warp and weft yarn axes, connecting the upper surface to the lower surface of the fabric (interlacing thread). This method is commonly referred to as the multistitched fabric structure [11]. The introduction of this configuration significantly influences the fiber volume fraction and permeability characteristics of the fabric [9]. In the design of the multilayer fabric structure, there exists an analytical transfer of threads, intricately interwoven vertically, traversing both the lower and upper surfaces of the fabric. This analytical thread transfer mechanism serves the purpose of enhancing the analytical flow to facilitate capillary-driven penetration of the fabric. The integration of this thread transfer analysis is executed as a distinct and separate process.

The production of the double-faced fabric in this research was accomplished using a Picanol GT-Max (rapier) 12 Dobby Weaving loom, with the motor operating at a set speed of 400 RPM (Figure 2d). The adjustment of the machine speed is intended to mitigate excessive engine vibrations that may lead to the failure of the weft thread launching process. This precaution is particularly crucial due to the more intricate woven structure employed in the production of this fabric, as compared to the simpler designs of plain weave or twill. Excessive vibrations in the machinery can induce variations in the tension of the yarn, and heightened yarn tension can adversely impact the quality of the resulting fabric. This impact includes the occurrence of deformations in the fabric and, consequently, the manifestation of defects such as holes in the produced fabric [8].

The fabrication process involved the utilization of 6600 warp threads, with a density of 98 warp threads per inch. The adjustment of variations in the number of weft yarn was determined by gradually reducing them by 10 strands, resulting in densities of 90 strands per inch, 80 strands per inch, and 70 strands per inch (Figure 2e). A low weft yarn density (below 70 strands per inch) would yield a porous fabric with high permeability, allowing the electrode paste to easily penetrate the fabric surface and causing short circuit during the application. Conversely, an elevated weft density exceeding 90 strands per inch may induce yarn breakage during the fabrication process. This occurrence is attributed to excessive tension applied during the shedding motion in the weaving machine. The double-faced fabric’s appearance can be seen in Figure 2f,g.

A woven fabric with a weft yarn count of 90 strands per inch exhibits a higher density compared to fabrics with 80 and 70 weft strands per inch. The density of the fabric is directly proportional to its weight but inversely proportional to its permeability [18]. Fabrics with 70 weft strands per inch demonstrate higher permeability in contrast to those with 80 and 90 weft strands per inch. Nevertheless, the fabric with 90 weft strands per inch possesses the highest mechanical properties due to its greater number of yarns, providing enhanced resistance against the tensile forces at play. Thus, while fabric density correlates with weight, it opposes permeability, and the mechanical strength of the fabric is maximized when employing a weft yarn count of 90 strands per inch [10].

## 3. Results and Discussion

### 3.1. Wetting Time and Spreading Speed Property of the Substrate

**Figure 3:**
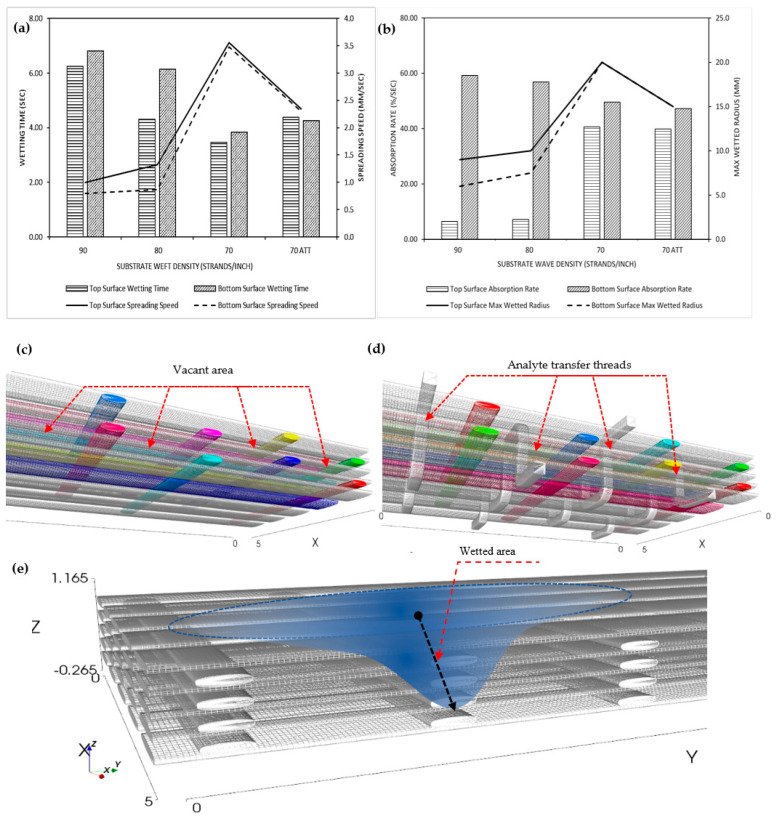
(**a**) Surface wetting time and surface spreading speed properties. (**b**) Surface absorption rate and surface maximum wetted radius properties. (**c**) Schematic 3D structural design of the double-faced woven substrate. (**d**) Schematic 3D structural design of the double-faced woven substrate with ATT. (**e**) Schematic 3D design of the fluid diffusion in double-faced woven substrate.

The results of the measurement of wetting time characteristics can be observed in Figure 3a. The measurement data indicate that the density of weft threads is directly proportional to the wetting time. As the number of constituent threads in a fabric increases, the required wetting time also increases. Double-faced fabric with a weft density of 70 strands per inch exhibits a faster wetting time compared to double-faced fabric with weft densities of 80 strands per inch and 90 strands per inch. This phenomenon can be attributed to the fact that the fluid flow on the fabric’s surface is directly influenced by the effectiveness of capillary pore distribution within the fabric [19,20]. Double-faced fabric with a weft density of 70 strands per inch has a lot of vacant area (porosity) when compared to other fabric structure designs, as can be seen in the schematic 3D structural design of the double-faced woven substrate in Figure 3c.

The porosity of the double-faced fabric with a weft density of 70 strands per inch decreases after the addition of the analyte transfer threads (ATT), as demonstrated in Figure 3d, thereby increasing the wetting time. This occurs because the analyte transfer thread configuration adds threads to the substrate fabric, increasing the number of threads per unit area and closing the capillary pores that were previously formed. The closure of these pores results in a reduction in the number of pores, ultimately leading to an increase in wetting time by 10–26% in double-faced fabric with a weft density of 70 strands per inch when the analyte transfer thread configuration is added. The configuration of interlacing threads, as described, serves to enhance the fluid diffusion from the upper surface of the fabric, where the working electrode is located, towards the lower surface, housing the reference electrode and counter electrode. Conversely, a similar diffusion process occurs in the reverse direction. This phenomenon arises due to the fluid’s ability to permeate through the air spaces between fibers within a thread and through the fibers composing the thread itself [13]. The diffusion process is further optimized by the choice of material for the interlacing thread, specifically rayon viscose, characterized by a lower twist, compared to cotton threads. This attribute ensures a more maximal air space between the fibers, thereby promoting optimal diffusion [16].

Figure 3a also depicts the graph of the measured spreading speed of fluid on the fabric surface; the double-faced fabric with a weft density of 70 strands per inch exhibits the fastest spreading speed compared to double-faced fabric with weft densities of 80 strands per inch and 90 strands per inch. There exists a correlation between the measured spreading speed of fluid on the fabric surface and the fabric density influenced by the density of the weft threads that compose it. The higher the weft thread density in a fabric, the fewer pores there will be in the fabric. This leads to an increase in the fabric’s cover factor [18]. An increased cover factor in a fabric results in an increase in hydrostatic resistance, which in turn leads to a decrease in the spreading speed of fluid on the fabric.

### 3.2. Absorption Rate and Maximum Wetted Radius of the Substrate

According to the terminology defined by AATCC (American Association of Textile Chemists and Colorists), the absorption rate is the average value of a fabric’s absorption capacity over a period of 20 s. The results of measuring the characteristics of the absorption rate are presented in Figure 3b. The absorption rate on the upper surface of the fabric shows a significant difference between fabrics with a weft thread density of 90 strands per inch and fabrics with a weft thread density of 70 strands per inch, with an increase of more than five times. The observed alteration in absorption can be attributed to a reduction in weft yarn density, decreasing from 90 strands per inch to 70 strands per inch, representing a nearly 30% decrement. Water absorption is intricately influenced by various factors, encompassing the fabric’s texture and yarn density. In this research, a lower weft yarn density results in a more relaxed top surface fabric structure, impacting the increase in voids between the fibers and, consequently, augmenting the fabric’s overall porosity. However, on the lower surface of the fabric, there is a decrease of 16.37% in the absorption rate for fabrics with a weft thread density of 70 strands per inch with ATT when compared to fabrics with a weft thread density of 70 strands per inch. The addition of the analyte transfer thread configuration to fabrics with a weft thread density of 70 strands per inch brings about relatively small changes, namely a decrease in the absorption rate ranging from 1.9% to 4.6%. Generally, the absorption rate on the lower surface of the fabric is always faster when compared to the absorption rate on the upper surface of the fabric [6]. This is because the fabric is placed horizontally during the measurement and gravity assists in increasing absorption on the lower surface of the fabric [4].

The measurement results indicate that the fabric with a weft thread density of 70 strands per inch achieves the highest maximum wetted radius on its surface, specifically with a value of 20 mm. The maximum wetted radius values for fabrics with weft thread densities of 90 and 80 strands per inch, respectively, fall within the ranges of 6–9 mm and 7.5–10 mm, as depicted in Figure 3b. The addition of the analyte transfer thread configuration results in a decrease in the maximum wetted radius of the fabric surface to 15 mm, but this value is relatively higher compared to the maximum wetted radius values of fabrics with weft thread densities of 90 and 80 strands per inch. Therefore, based on the measurement data, it can be concluded that the fabric with a weft thread density of 70 strands per inch exhibits the widest horizontal spreading range (along the x and y axes of the fabric).

### 3.3. Accumulative One-Way Transport Index of the Substrate

Based on measurements of wetting time characteristics, fluid spreading rate on the fabric surface, absorption rate, and maximum horizontal wetted radius of the fabric surface, it can be generally concluded that fabrics with a weft thread density of 70 strands per inch and the fabrics with a weft thread density of 70 strands per inch with ATT are the most suitable variation in this research. However, it is necessary to analyze the fabric’s ability to vertically diffuse fluid from one fabric surface to another (Axis—Z), as depicted in Figure 3e. This can be determined through the measurement of the accumulative one-way transport index, which is a crucial characteristic in the development of biosensors with a multilayer concept, where electrodes are positioned on two different fabric surfaces. Therefore, the substrate used must have the capability to efficiently and abundantly transport fluid from one fabric surface to another as quickly as possible, following the capillary-driven fluid propagation along the fabric’s thread axis [21].

**Figure 4:**
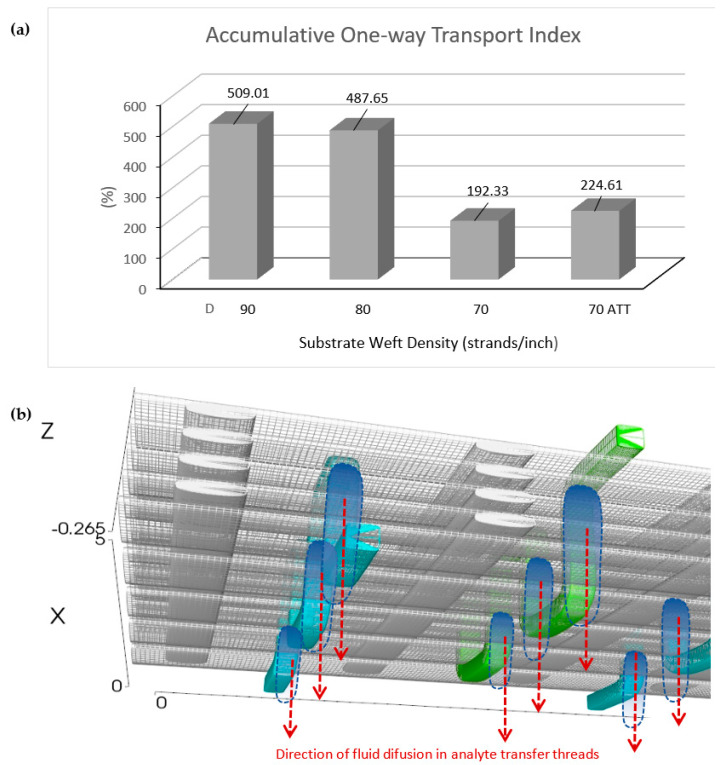
(**a**) Accumulative one-way transport index of the substrate. (**b**) The mechanism of fluid flow in fabric with a weft thread density of 70 strands per inch ATT.

The measurements of the accumulative one-way transport index indicate that fabrics with a weft thread density of 70 strands per inch have the lowest values compared to fabrics with weft thread densities of 90 and 80 strands per inch, as shown in Figure 4a. Therefore, even though fabrics with a weft thread density of 70 strands per inch exhibit the widest horizontal spreading range, this variation has the lowest fluid transport capability. Fabrics with weft thread densities of 90 and 80 strands per inch have higher weft thread densities, leading to higher fluid flow rates [22].

The addition of an analytical configuration of transfer threads to a fabric with a weft thread density of 70 threads per inch results in a cumulative increase of 16.8% in the one-way fluid flow index. This enhancement is attributed to the ability of fluid to diffuse through the hydrophilic constituent fibers of the analytical transfer thread, which contains -OH, C=O, and -CO_2_R groups. This condition facilitates transient hydrogen bonding with water molecules, thereby initiating the diffusion mechanism within the analyte transfer threads’ configuration [23]. Within the analyte transfer threads’ configuration, there are threads positioned perpendicular to both the warp and weft axes, vertically penetrating the upper and lower surfaces of the fabric. This structural arrangement promotes fluid flow from one surface to another due to the spread or propagation of fluid through the capillary spaces present between the fibers within the analyte transfer thread configuration [24]. The mechanism of fluid flow in a fabric with a weft thread density of 70 threads per inch, with the addition of an analyte transfer thread configuration, is illustrated in Figure 4b. The characteristics of a fabric with a cumulative one-way fluid flow index are of paramount importance in applications that rely on fluid flow or moisture transport principles [12].

### 3.4. Finger Print of Moisture Management Properties and Classification of the Substrate

**Figure 5:**
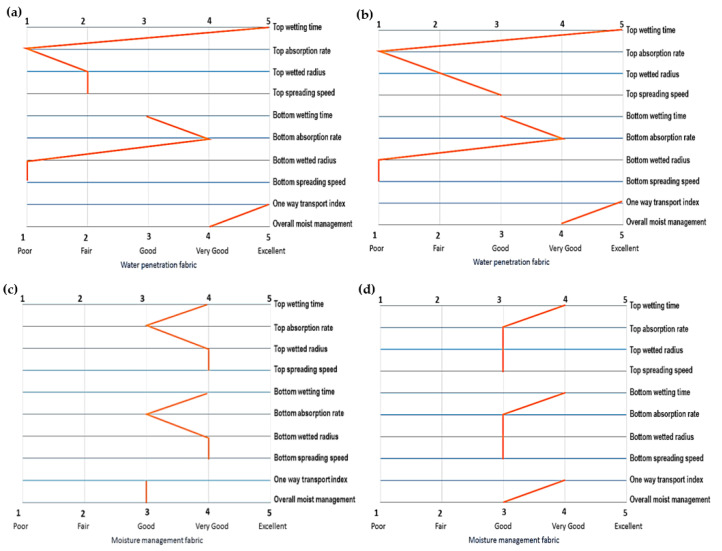
Finger print of moisture management properties: (**a**) 90 strands per inch; (**b**) 80 strands per inch; (**c**) 70 strands per inch; (**d**) 70 strands per inch with analyte transfer thread.

All quantitative data obtained from prior measurements underwent analysis in accordance with the AATCC Test Method 195-2009 [17]. Subsequently, the data were categorized on a scale ranging from one to five and organized into a specific arrangement resembling a fingerprint. The fingerprint of the moisture management properties of the substrates are shown in Figure 5a–d. From the dataset analysis, it becomes apparent that among the various weft thread density configurations examined, only two specific densities fall within the category designated as “Moisture Management Fabric”. These densities are characterized by weft thread counts of 70 strands per inch and 70 strands per inch with ATT. Within the context of fabrics classified as “Moisture Management Fabric”, it is observed that the surface wetting process by fluids is significantly improved, facilitating fluid penetration through the fabric’s thickness, effectively traversing from one surface to another [23].

## 4. Conclusions

According to the analysis using the AATCC method, it has been determined that fabrics manufactured with a specific structural design, utilizing polyester-viscose fibres material and a weft thread density of 70 strands per inch are capable of meeting the classification of moisture management fabric. In the context of this research, the most suitable fabric to serve as a substrate for a non-invasive sensor with a multilayer concept is the one with a weft thread density of 70 strands per inch, supplemented with an analyte transfer thread configuration. It is essential to note that the fluid requires a certain amount of time to reach the peak of wetting and absorption of the fabric during the biosensor application process.

Therefore, further investigation is warranted to determine the specific duration required for this process. This can be accomplished by analyzing the data obtained from measuring the relationship between the moisture content in the fabric and time, utilizing fabric substrates with a weft thread density of 70 strands per inch and an additional analyte transfer thread configuration. This analysis will provide valuable insights into the kinetics of fluid interaction with the fabric, which are crucial for optimizing the performance of the non-invasive biosensor.

## Data Availability

The data presented in this study are available on request from the corresponding author. The data are not publicly available due to internal policy.
